# Synergy between PEDF and Doxorubicin in Breast Cancer Cells: Effects on Metastatic and Metabolic Pathways

**DOI:** 10.3390/ijms25052755

**Published:** 2024-02-27

**Authors:** Raziyeh Abooshahab, Hani Al-Salami, Crispin R. Dass

**Affiliations:** 1 Curtin Medical School, Curtin University, Bentley 6102, Australia; 2 Curtin Health Innovation Research Institute, Bentley 6102, Australia; 3 Biotechnology and Drug Development Research Laboratory, Curtin Health Innovation Research Institute, Bentley 6102, Australia

**Keywords:** breast cancer, combination treatment, Dox, PEDF, metastasis, signaling pathways

## Abstract

Pigment epithelium-derived factor (PEDF), a serine protease inhibitor (Serpin) family member, shows promise in inhibiting tumour growth. In our study, we explored the effects of PEDF on the efficacy of the frontline chemotherapy agent doxorubicin (Dox) in BC cells. We found that Dox+PEDF treatment significantly reduced glucose uptake in MDA-MB-231 cells compared to the control (*p* = 0.0005), PEDF (*p* = 0.0137), and Dox (*p* = 0.0171) alone but paradoxically increased it in MCF-7 cells. Our findings further revealed that PEDF, Dox, and Dox+PEDF substantially hindered tumour cell migration from tumour spheroids, with Dox+PEDF showing the most significant impact (*p* < 0.0001). We also observed notable decreases in the expression of metastatic markers (uPAR, uPA, CXCR4, MT1-MMP, TNF-α) across all treatment groups (*p* < 0.0001) in both cell lines. When it comes to metabolic pathways, PEDF increased phosphorylated IRS-1 (p-IRS1) levels in MDA-MB-231 and MCF-7 (*p* < 0.0001), while Dox decreased it, and the combination led to an increase. In MDA-MB-231 cells, treatment with PEDF, Dox, and the combination led to a notable decrease in both phosphorylated AKT (p-AKT) and total AKT levels. In MCF-7, while PEDF, Dox, and their combination led to a reduction in p-AKT, total levels of AKT increased in the presence of Dox and Dox+PEDF. Combining PEDF with Dox enhances the targeting of metastatic and metabolic pathways in breast cancer cell lines. This synergy, marked by PEDF’s increasing roles in cancer control, may pave the way for more effective cancer treatments.

## 1. Introduction

Breast cancer (BC), a complex and multifaceted disease, remains one of the leading causes of cancer-related deaths among women worldwide [1]. It is categorised into various types depending on the presence or absence of certain receptors [2]. These include the oestrogen receptor (ER), progesterone receptor (PR), and human epidermal growth factor receptor 2 (HER2), leading to the classification of BC into specific subtypes: the luminal subtype (ER/PR positive); the HER2 positive subtype (with an overexpression of the HER2 receptor); and the triple-negative or basal-like subtype (TNBC), which lacks all these receptors [3]. Despite recent advances in oncological research, the search for more effective treatments continues, as the heterogeneity of BC often leads to resistance and relapse following conventional therapies.

Central to the progression and treatment resistance in BC is the intricate network involving pathways like the mitogen-activated protein kinases (MAPKs) and the PI3K/Akt pathways [4,5]. The PI3K/Akt signalling pathway operates under stringent control, ensuring the proper regulation of cellular processes. However, in the context of cancer, this pathway can become dysregulated, which may arise from mutations in the genes that encode the components of the pathway, the loss of function in tumour suppressor genes that typically serve to restrain the pathway, or the irregular expression of regulatory proteins [6,7]. Such dysregulation plays a pivotal role in cancer development and progression, contributing to hallmark cancer traits, including un-checked cell proliferation, aggressive tumour behaviour, alterations in cellular metabolism favouring tumour growth, and the development of resistance to therapies [5,6]. Therefore, this pathway is of great importance towards understanding the initiation and progression of cancer, its value in the prognosis and diagnosis of BC patients, and its potential as a therapeutic target.

In the ongoing battle against BC, combination therapies have emerged as a promising strategy, potentially overcoming the limitations of single-agent treatments [8]. Among these, the combination of doxorubicin (Dox), a well-established and frontline chemotherapeutic, and pigment epithelium-derived factor (PEDF), a naturally occurring protein with potent anti-angiogenic properties, represents a novel and intriguing approach. Dox, known for its efficacy in a broad range of cancers, functions primarily by intercalating DNA and inhibiting topoisomerase II, leading to apoptosis in uncontrolled dividing cells [9]. However, its clinical use is often limited by systemic toxicity and the development of drug resistance. On the other hand, PEDF, initially identified as a neurotrophic factor, has gained attention for its anti-metastatic and anti-angiogenic effects [9,10]. These functions of PEDF not only inhibit tumour growth but also disrupt the tumour microenvironment, which is critical for cancer progression [10].

PEDF may mitigate some of the adverse effects of Dox. Earlier studies by our group have demonstrated that PEDF can reduce the adverse effects of Dox on critical organs, including the heart, small intestine, and testes [11]. Herein, we explore the individual roles of Dox and PEDF and unveil the mechanism behind their synergistic potential in two BC cells. MDA-MB-231, a TNBC cell line, lacks ER, PR, and HER2 expressions. This subtype is characterised by aggressive behaviour and a lack of targeted hormone therapies. In contrast, MCF-7 cells are ER-positive and more responsive to hormonal therapies, reflecting a different BC subtype. These inherent differences significantly influence their responses to treatments like PEDF and Dox. In an effort to substantiate the merits of this combination, our focus was placed on the interrogation of key molecular markers that are pivotal in the PI3K/Akt signalling pathway and metastatic potential.

## 2. Results

### 2.1. Effects of PEDF, Dox, and Their Combination on Cell Viability

**Figure 1:**
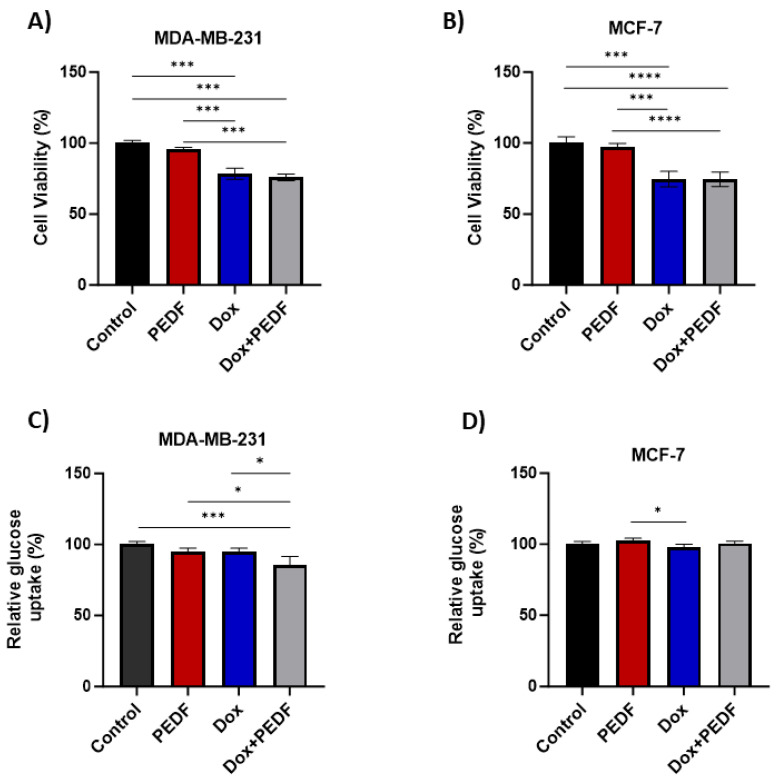
CellTitre Blue assay was used to determine the viability of (**A**) MDA-MB-231 and (**B**) MCF-7 exposed to PEDF (100 nM), Dox (0.5 µM), and Dox+PEDF. (**C**,**D**) Glucose uptake was further measured in MDA-MB-231 and MCF-7 following PEDF, Dox, and Dox+PEDF. PEDF, pigment epithelium-derived factor; Dox, doxorubicin. n = 4; (*) *p* ≤ 0.05; (***) *p* ≤ 0.001, and (****) *p* ≤ 0.0001.

Our study investigated the effects of PEDF and Dox, both separately and in combination, on the viability of MDA-MB-231 and MCF-7 cancer cell lines. In the MDA-MB-231 BC cell line, exposure to PEDF alone resulted in a slight decrease in cell viability, but this was not significant (*p* > 0.05) (Figure 1A). As anticipated, treatment with Dox led to a significant decrease in cell viability compared to control and PEDF (*p* < 0.0001), indicating a pronounced cytotoxic effect on MDA-MB-231 cells. Akin to Dox, the combination treatment showed a decrease in viability compared to the control (*p* < 0.0001), and these changes were also evident when compared to PEDF (*p* < 0.0001). Similarly, in the MCF-7 breast cancer cell line (Figure 1B), the pattern of response to PEDF, Dox, and their combination was consistent with that observed in MDA-MB-231 cells. PEDF alone did not significantly affect the viability of MCF-7 cells (*p* > 0.05). However, Dox treatment resulted in a considerable reduction in cell viability compared to control (*p* < 0.0001) and PEDF (*p* < 0.0001). Furthermore, combining Dox and PEDF similarly to the Dox alone led to a decrease in cell viability (control vs. Dox+PEDF and Dox+PEDF vs. PEDF; *p* < 0.0001), showing no effect of PEDF on cell viability, either alone or in combination with Dox. These findings suggest that while Dox significantly decreases cell viability, PEDF does not influence cell viability on its own, nor does it alter the efficacy of Dox when used in combination. This outcome highlights the potent cytotoxic effect of Dox, irrespective of the presence of PEDF.

### 2.2. Effects of PEDF, Dox, and Their Combination on Glucose Uptake

This study explored the effects of PEDF and Dox, individually and in combination, on glucose uptake in BC cell lines. For MDA-MB-231, treatment with PEDF alone decreased glucose uptake, but this change was not statistically significant (*p* > 0.05). Similarly, exposure to Dox reduced glucose uptake, yet this effect did not reach statistical significance (*p* > 0.05). However, the combination of PEDF and Dox exhibited a significant decrease in glucose uptake in MDA-MB-231 cells compared to control (*p* = 0.0005) (Figure 1C). This decrease was also seen compared to the PEDF (Dox+PEDF vs. PEDF; *p* = 0.0137) and Dox (Dox+PEDF vs. Dox; *p* = 0.0171) groups. This suggests a synergistic effect of these two agents in altering glucose metabolism, impacting the cell’s energy utilisation.

Notably, contrasting responses were observed in the MCF-7 cell line (Figure 1D). Exposure to PEDF alone resulted in a slight increase in glucose uptake, although this was not statistically significant (*p* > 0.05). When treated with Dox, MCF-7 cells showed a decrease in glucose uptake similar to MDA-MB-231. There was no difference between PEDF and combined treatment (Dox+PEDF). This may indicate a differential cellular response to the combined treatment, possibly reflecting distinct metabolic adaptations between the two cell lines and/or different functions of PEDF in different breast tumour environments.

### 2.3. Effects of PEDF, Dox, and Their Combination on Tumour Cell Migration

**Figure 2:**
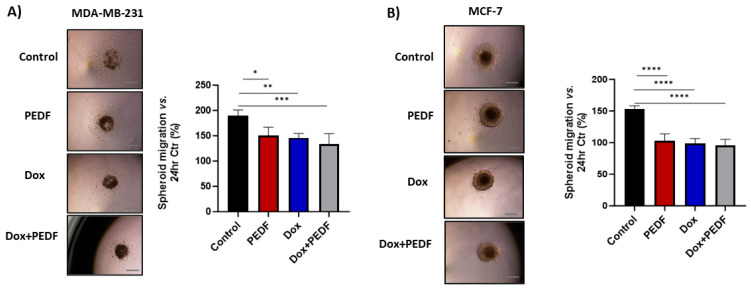
Inhibition of cancer cell migration by PEDF (100 nM), Dox (0.5 µM), and Dox+PEDF treatments in (**A**) MDA-MB231 and (**B**) MCF-7. The migration of tumour cells was recorded by inverted microscope: scale bar, 50 μm. The migration areas were measured and presented as mean area changes relative to the areas at 24 h. PEDF, pigment epithelium-derived factor; Dox, doxorubicin; Ctr, control. n = 4; (*) *p* ≤ 0.05; (**) *p* ≤ 0.01; (***) *p* ≤ 0.001, and (****) *p* ≤ 0.0001. Scale bar = 50 µM.

The migration of tumour cells assay was based on a tumour spheroid model. As shown in Figure 2A, MDA-MB-231 tumour cells in the control group exhibited a rapid migration along the plate away from tumour spheroids after 24 h. In contrast, the migration of tumour cells was dramatically inhibited by 24.47%, 23.13%, and 29.59% after tumour spheroids were treated with PEDF (*p* = 0.0144), Dox (*p* = 0.0063), and Dox+PEDF (*p* = 0.0009), respectively. While the combination of Dox+PEDF showed more reduction in cell migration, these differences were not significant between treated groups. Similarly, in the MCF-7 spheroid cultures, there was a notable reduction in migration distance in all three treatment groups by 32.60% for PEDF (*p* < 0.0001), 35.72% for Dox (*p* < 0.0001), and 37.28% for Dox+PEDF (*p* < 0.0001) when compared to the control (Figure 2B). Although Dox+PEDF showed a noticeable effect on inhibiting tumour migration, there were no significant differences between treatment groups.

### 2.4. Effects of PEDF, Dox, and Their Combination on Metastatic Markers

**Figure 3:**
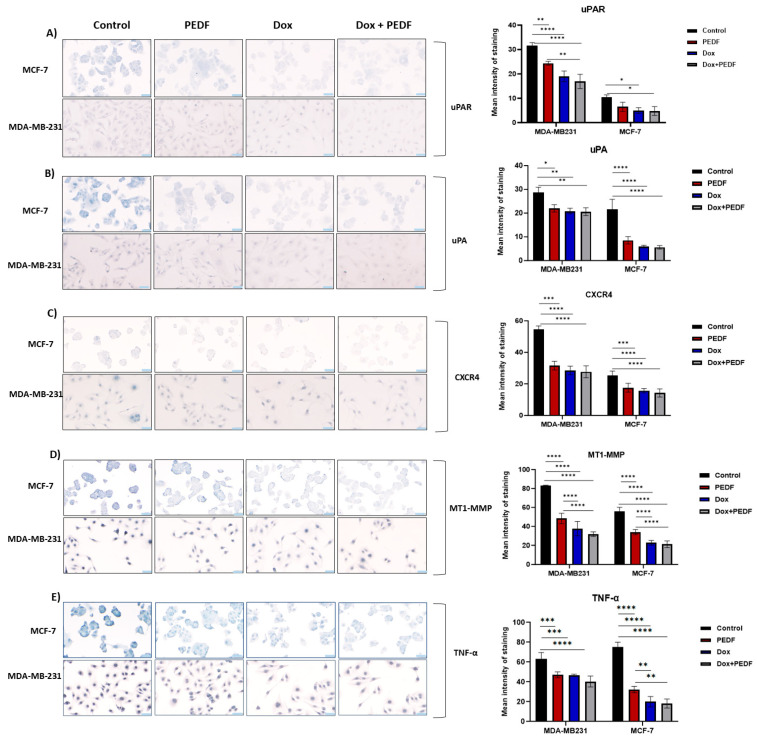
Metastatic markers expression via immunocytochemistry staining (blue) in MCF-7 and MDA-MB-231 cells following exposure to PEDF (100 nM), Dox (0.5 µM), and Dox+PEDF treatments, including (**A**) uPA, (**B**) uPAR, (**C**) CXCR4, (**D**) MT1-MMP, and (**E**) TNF-α. Graphs show the mean intensity of staining (blue). PEDF, pigment epithelium-derived factor; Dox, doxorubicin; uPA, urokinase-type plasminogen activator; uPAR, urokinase-type plasminogen activator receptor; CXCR4, C-X-C motif chemokine receptor 4; MT1-MMP, membrane-type 1 matrix metalloproteinase; TNFα, tumour necrosis factor. Significant differences were calculated using Tukey’s multiple compression tests and indicated as (*) *p* ≤ 0.05, (**) *p* ≤ 0.01, (***) *p* ≤ 0.001, and (****) *p* ≤ 0.0001. n = 4. Scale bar = 50 µM.

Based on the above findings, we next examined metastatic markers. In the context of urokinase-type plasminogen activator receptor (uPAR) expression, significant immunocytochemistry (ICC) findings emerged (Figure 3A). For MDA-MB-231 cells, we observed notable reductions across all treatment groups compared to the control, with the most pronounced effects seen with Dox and Dox+PEDF (*p* < 0.0001). Importantly, a combination of Dox+PEDF revealed a significant reduction compared to PEDF alone (*p* < 0.007). In MCF-7 cells, the impact was slightly different. Here, Dox and Dox+PEDF treatments led to significant reductions in uPAR expression, contrasting with the negligible change induced by PEDF alone (*p* > 0.05). For urokinase-type plasminogen activator (uPA) expression (Figure 3B) in MDA-MB-231 cells, all treatments, including PEDF, Dox, and Dox+PEDF, resulted in significant decreases compared to the control group. However, no significant differences were noted among the treated groups, indicating a similar level of efficacy (*p* > 0.05). In the MCF-7 cell line, this trend persisted. All treatments significantly reduced uPA expression (*p* < 0.0001), again with no notable differences observed among the treatment groups (*p* > 0.05). Turning our attention to C-X-C motif chemokine receptor 4 (CXCR4) expression, a parallel response was evident in both cell lines (Figure 3C). In MDA-MB231 cells, all treatment groups showed significant decreases in CXCR4 expression compared to the control (*p* < 0.0001), with no significant variances between the different treatments (*p* > 0.05). This uniform response was mirrored in the MCF-7 cells, where treatments with PEDF, Dox, and Dox+PEDF all significantly lowered CXCR4 expression (*p* < 0.0001), maintaining a consistent pattern across the treatment spectrum, with no differences among treated groups (*p* > 0.05). Examining membrane-type 1 matrix metalloproteinase (MT1-MMP) expression, we observed a similar trend in the MDA-MB231 cell line (Figure 3D), where all treatments led to significant reductions (*p* < 0.0001). Notably, the combination of PEDF and Dox exhibited a pronounced effect compared to PEDF alone (*p* < 0.0001). In MCF-7 cells, this pattern of significant decreases across all treatment groups was again evident, particularly in Dox+PEDF vs. PEDF (*p* < 0.0001), although no significant distinctions were found between the Dox and Dox+PEDF groups (*p* > 0.05). In the context of tumour necrosis factor α (TNFα), the most significant reduction was seen in Dox+PEDF (*p* < 0.0001), although all treatments demonstrated substantially lower TNFα levels in MDA-MB-231. In MCF-7, TNFα levels were comparably reduced by all treatments, with Dox showing a similar level of effectiveness alone or in combination with PEDF (*p* < 0.0001) (Figure 3E). The detailed changes among groups are presented in Table 1.

### 2.5. Modulation of Metabolic Markers by PEDF, Dox, and Their Combination

**Figure 4:**
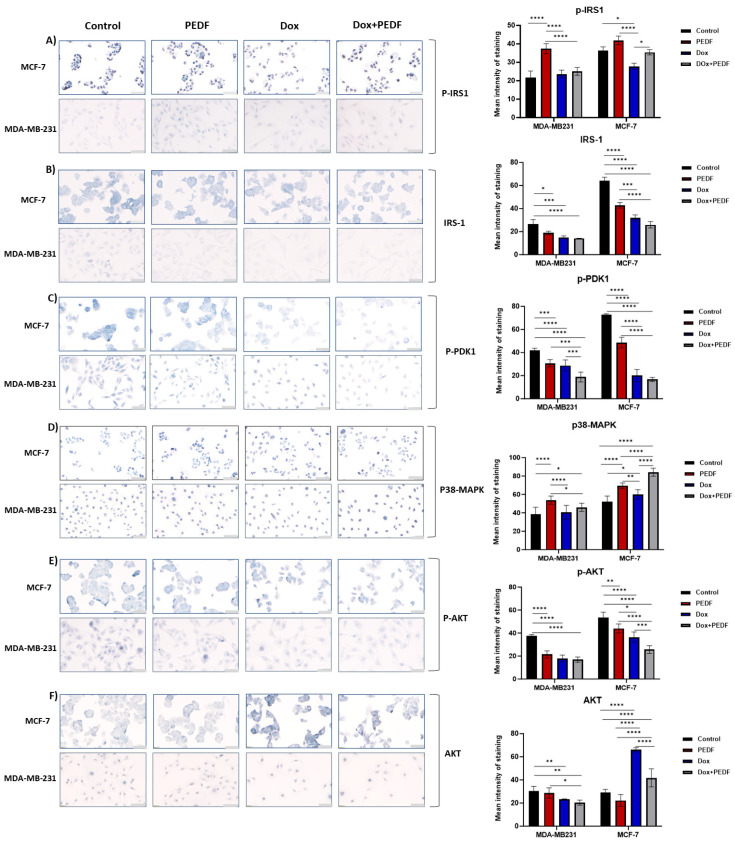
Cellular signalling markers expression via immunocytochemistry staining (blue) in MCF-7 and MDA-MB-231 following exposure to PEDF (100 nM), Dox (0.5 µM), and Dox+PEDF treatments, including (**A**) p-IRS1, (**B**) IRS-1, (**C**) p-PDK1, (**D**) p38-MAPK, (**E**) p-AKT, and (**F**) AKT. Graphs show the mean intensity of staining (blue). PEDF, pigment epithelium-derived factor; Dox, doxorubicin; p-IRS-1, IRS-1, insulin receptor substrate 1; PDK1, phosphoinositide-dependent kinase 1; p38-MAPK, p38 mitogen-activated protein kinases; AKT, serine/threonine kinase. Significant differences were calculated using Tukey’s multiple compression tests and indicated as (*) *p* ≤ 0.05, (**) *p* ≤ 0.01, (***) *p* ≤ 0.001, and (****) *p* ≤ 0.0001. n = 4. Scale bar = 50 µM.

As shown in Figure 4A, in MDA-MB-231 cells, PEDF treatment markedly increased insulin receptor substrate 1 phosphorylated form (p-IRS1) levels (*p* < 0.0001). Slight increases were observed in Dox and combination treatments, but these changes were not significant (*p* > 0.05), suggesting a unique mechanism of action for PEDF. For insulin receptor substrate 1 (IRS-1), we found substantial decreases across all treatment groups (Figure 4B), with Dox+PEDF presenting a more pronounced reduction (Dox+PEDF vs. control, *p* = 0.0001). This shift in signalling intensity was also reflected in the phosphorylated phosphoinositide-dependent kinase 1 (p-PDK1) marker (Figure 4C), where all treatments achieved significant declines, mostly in the combination group (*p* < 0.000), implying a widespread treatment effect on this kinase. Moreover, these reductions were more prominent between Dox+PEDF vs. PEDF (*p* = 0.0002) and Dox+PEDF vs. Dox (*p* = 0.0023), emphasising a higher effect on this marker when PEDF and Dox were combined. Interestingly, p38 mitogen-activated protein kinase (p38-MAPK) levels were significantly attenuated by PEDF compared to control (*p* < 0.0001), while Dox+PEDF exerted a more moderate influence (*p* = 0.02820), indicating a differential response to combination therapy (Figure 4D). In addition, the changes in p38-MAPK levels were more significant between PEDF and Dox (*p* < 0.0001); however, all treatments led to an increase in the level of this marker. The response pattern continued with serine/threonine kinase phosphorylated form (p-AKT), where all treatments led to considerable decreases compared to control (*p* < 0.0001), highlighting their collective impact on AKT phosphorylation (Figure 4E). However, there were no significant changes between PEDF and Dox alone in regard to Dox+PEDF (*p* > 0.05). According to Figure 4F, the total serine/threonine kinase (AKT) marker decreased significantly with Dox (*p* = 0.0367) and the combined Dox+PEDF (*p* = 0.0016) treatments, suggesting a synergistic effect on AKT expression. Furthermore, a significant difference between PEDF and Dox+PEDF was observed (*p* = 0.0140).

The MCF-7 cell line exhibited different treatment responses across the signalling markers. Similar to MDA-MB-231, PEDF treatments led to an increment in p-IRS1 staining (Figure 4A), but the changes were not significant (*p* > 0.05). This effect was not paralleled by Dox but by the combination treatment. Treatment with Dox led to a considerable decrease in p-IRS1 (*p* = 0.0083), and the combination treatment led to an increase when compared to Dox (*p* < 0.0001) but not PEDF alone (*p* > 0.05). This may suggest a cell line-specific sensitivity to both PEDF and Dox and a modulation effect of PEDF on Dox function. The uniform response extended to IRS-1 (Figure 4B), where the staining intensities were notably decreased across all treatments (*p* < 0.0001). Moreover, these significant decreases were observed between PEDF and Dox (*p* = 0.0003) and Dox+PEDF vs. PEDF (*p* < 0.0001). Similarly, for p-PDK1, the treatments reduced the expression (*p* < 0.0001), with the most striking changes observed between PEDF and Dox+PEDF (*p* < 0.0001) (Figure 4C). When examining p38-MAPK, Dox+PEDF notably increased the staining intensity compared to the control (*p* < 0.0001) (Figure 4D), again reflecting a potential additive effect of the combination treatment. Remarkable changes were also observed between PEDF and Dox separately compared to Dox+PEDF. The p-AKT followed suit, with all treatments inducing significant reductions, yet the combinatorial treatment showed the most marked effect, indicating an enhanced efficacy (Control vs. Dox+PEDF; *p* < 0.0001), with notable changes between combination therapy and PEDF and Dox alone (Figure 4E). Total AKT levels (Figure 4F) contrasted this trend with substantial treatment-induced increases, particularly evident in the Dox group (*p* < 0.0001). Furthermore, the PEDF group showed a decrease in the level of AKT (control vs. PEDF; *p* = 0.0437). The data presented in Table 2 indicate a potential synergistic or modulatory effect of PEDF on the functionality of Dox-influenced environments.

## 3. Discussion

The intricate role of cell signal transduction in the development and progression of BC represents a complex and multifaceted domain of scientific inquiry. At the core of this is the activation of the MAPKs and the PI3K/Akt pathways, recognised for their critical input into initiating tumour metabolic reprogramming, fostering the Warburg effect, processes related to metastasis, disease progression, and the development of resistance to therapeutic interventions in breast tumours [12,13]. In this complex scenario, the strategic implementation of combination therapy, particularly those with reduced side-effect profiles, assumes a critical significance. PEDF, encoded by the *SERPINF1* gene, belongs to the serpin (serine protease inhibitor) superfamily of proteins, which is a multifunctional protein known for its diverse roles, including differentiation, neuroprotection, anti-angiogenesis, anti-apoptosis, and anti-metastasis [10]. Crucially, several studies have highlighted a consistent downregulation of PEDF expression in BC, with a marked decrease observed particularly in metastatic BC cells [14,15], highlighting its potential significance in tumour progression and metastasis. Recent evidence has highlighted an intriguing interaction between PEDF and Dox, a frontline anticancer drug known for its efficacy against various cancers, including osteosarcoma [11]. Notably, PEDF has been observed to alleviate the toxicity of Dox in sensitive tissues such as the heart, small intestine, and testis [11]. Furthermore, in human BC cell lines, increased PEDF levels were noted with escalating doses of Dox, indicating a reciprocal regulatory relationship between PEDF and Dox [16]. However, the exact mechanism by which PEDF influences the action of Dox, and vice versa, warrants further elucidation. Given these insights, our research interest pivots on exploring whether the concomitant administration of PEDF with chemotherapeutic agents like Dox could yield synergistic effects, not only in inhibiting tumour growth but also in modulating critical pathways such as MAPK and PI3K/AKT signalling pathways and metastasis in BC.

Our observation of Dox significantly reducing cell viability in MDA-MB-231 and MCF-7 cell lines aligns with the existing research [17,18], highlighting its efficacy as a chemotherapeutic agent. Similar cytotoxicity was observed with the combination of Dox and PEDF in MDA-MB-231 and MCF-7 compared with the cells treated with Dox alone, suggesting no effect of PEDF on viability and/or synergistic effects of the combination on cells’ survival. The main molecular pathways involved in controlling aerobic glycolysis are the PI3K/AKT and AMPK pathways [19], leading to increased glucose uptake. In our study, the significant decrease in glucose uptake in MDA-MB-231 cells, especially with combined Dox treatment, indicates a disruption in their metabolic pathways. In contrast, the slight increase in glucose uptake in MCF-7 cells, particularly with PEDF treatment, might reflect the metabolic flexibility associated with ER-positive breast cancer cells, where hormonal signals intersect with the metabolic regulation [20]. Dox alone led to reduced glucose uptake; however, when combined with PEDF, there was an increase in glucose uptake again in MCF-7 cells, suggesting a modulation effect of PEDF on the function of Dox in different environments. TNBC cells, like MDA-MB-231, typically have upregulated metabolic pathways to support their energy requirements for rapid growth [21]. This elevated metabolic requirement is commonly satisfied through increased glucose uptake, a phenomenon extensively understood through the lens of the Warburg effect [22]. Our latest findings have highlighted that markedly impacts this metabolic reprogramming by PEDF, particularly the Warburg effect [23]; comparing them with the current results can validate the usefulness of PEDF in modulating metabolism in TNBC.

In BC, IRS-1 and its phosphorylated form (p-IRS1) are key mediators in the insulin and insulin-like growth factor (IGF) signalling pathways and play crucial roles in the cellular signalling pathways influencing tumour progression and treatment response [24]. When IRS-1 is phosphorylated, it activates various downstream signalling cascades, including the PI3K/AKT pathway, which is vital for cell survival, proliferation, and metabolism [25]. Perturbation in this activation can enhance tumour growth and resistance to certain therapies. Additionally, the expression levels of IRS-1 and its phosphorylated state can serve as potential biomarkers for BC prognosis and may guide therapeutic strategies [24]. In our study, both cell lines exhibited somewhat different responses, possibly due to their altered metabolic demands. PEDF’s effect in increasing p-IRS1 levels, especially in MDA-MB-231 cells, may suggest a shift towards altered insulin sensitivity, while Dox potentially disrupts these pathways, contributing to decreased cell survival. On the other hand, combination therapy showed an increase in the level of p-IRS1 when compared with Dox treatment alone, which might suggest an antagonistic interaction between PEDF and Dox. A decrease in the total IRS-1 levels in all group treatments, specifically PEDF treatment, could indicate a negative feedback loop or a shift in the cellular signalling dynamics. It might suggest that while PEDF activates IRS-1 signalling initially (as evidenced by increased p-IRS1), it concurrently triggers mechanisms that reduce the overall abundance of IRS-1, possibly to control or limit the signal transduction.

In the MDA-MB-231 context, treatment with PEDF, Dox, and the combination of both (Dox+PEDF) led to a substantial decrease in both phosphorylated AKT (p-AKT) and total AKT levels, underlying a decrease in the overall quantity of the protein available for signalling and activation [25]. The fact that there was no significant difference within the treated groups implies that each treatment, whether individually or in combination, effectively downregulated AKT signalling in these cells. The increase in p-IRS1 and the decrease in total IRS-1 levels, along with a decrease in both p-AKT and total AKT levels, suggest that while PEDF initially activates the IRS-1 signalling (increased p-IRS1), it also triggers a feedback mechanism that inhibits the downstream PI3K/Akt pathway (decreased p-AKT and AKT). Dox alone led to an increase in AKT in MCF-7 cells, which is notable. Despite a decrease in phosphorylated form, the increased expression of total AKT in response to Dox and the combination treatment could be interpreted as a cellular compensatory mechanism. However, the increase in AKT when Dox was combined with PEDF was less than Dox alone, which could be explained by a complex interaction between the cytotoxic effects of Dox and the signalling modulation by PEDF. It is possible that PEDF’s influence on other pathways (like MAPK) [26] or its impact on cellular stress responses act to modulate the activation of AKT by Dox in this context. In addition, MCF-7 cells might be upregulating total AKT production in an attempt to overcome the inhibition of the phosphorylated form. This phenomenon is often observed in cancer cells, where drug treatments lead to adaptive responses [27].

p-PDK1 is also crucial in the PI3K/AKT signalling pathway, a key regulator of cell growth, proliferation, and survival. Abnormal activation of this protein is often observed in BC and is associated with aggressive tumour characteristics and poor prognosis [28]. In our study, p-PDK1 downregulation indicates suppressed PI3K/AKT signalling, which is crucial for survival and proliferation. The response in MCF-7 cells may be more marked, aligning with their dependency on hormones and not being aggressive. The combined treatment effectively reduces p-PDK1 levels in both cell lines, suggesting a comprehensive impact on cell survival mechanisms.

p38-MAPK is involved in cellular responses to stress, including oxidative stress and DNA damage [29], and has been shown to mediate both tumour suppression and tumour promotion in a context-dependent manner [30]. In our study, PEDF, Dox, and a combination of the two increased p38-MAPK expression in both cell lines. PEDF might modulate the expression of this protein differently, contributing to reduced cellular stress responses and enhancing the efficacy of Dox in inducing apoptosis. p38-MAPK can interact with other signalling pathways, including PI3K/Akt and the MAPK/ERK pathway [30]. For instance, activation of p38-MAPK can lead to the inhibition of the PI3K/Akt pathway [31], which could contribute to the observed decrease in p-AKT and AKT levels. The combination of PEDF and Dox may create a unique cellular environment where stress responses and signalling pathway modulations are both enhanced, leading to a more pronounced activation of p38-MAPK.

The observed reduction in tumour cell migration is consistent with previous findings on the anti-metastatic potential of Dox [17]. However, the contribution of PEDF to this process, especially in combination with Dox, is less documented [10]. This study investigates the influence of PEDF, Dox, and their combination on spheroid-based migration. All treatments showed the same drastic decrease in migration of cells after 24 h compared to the control with no synergistic effect of the combination. Moreover, our findings on the modulation of metastatic markers such as uPAR, uPA, CXCR4, MT1-MMP, and TNF-α by PEDF, Dox, and their combinations add critical insights into the molecular mechanisms underlying cancer progression and confirmed the results from migration. All markers showed the same response pattern after exposure to the treatments, with no synergy or cross-talk between PEDF and Dox when combined. The observed decrease was more pronounced in MT1-MMP in both cell lines, underlining the treatment’s efficacy in inhibiting invasion. However, the impact was more evident in MDA-MB-231 cells due to their heightened invasive nature. Dox’s role in downregulating MT1-MMP could be complemented by PEDF’s anti-angiogenic activity, which collectively results in reduced invasiveness.

**Figure 5:**
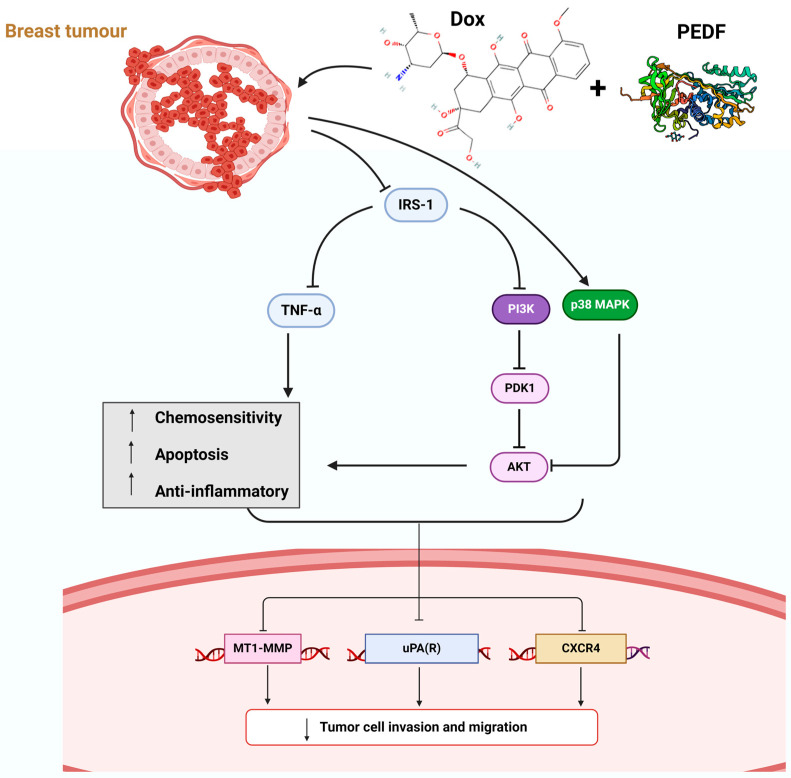
A schematic representation of the therapeutic effects of combining Dox+PEDF in BC treatment. It illustrates the pathway modifications induced by this combination strategy focusing on MAPK and PI3K/Akt pathways, which are important for cell growth and survival, through adjustments in the levels of IRS, PDK1, p38-MAPK, and p-AKT, which ultimately leads to reducing the levels of certain proteins (uPAR, uPA, CXCR4, TNF-α, and MT1-MMP) on stopping cancer spread. BC, breast cancer; PEDF, pigment epithelium-derived factor; Dox, doxorubicin; IRS-1, insulin receptor substrate 1; PDK1, phosphoinositide-dependent kinase 1; p38MAPK, p38 mitogen-activated protein kinases; AKT, serine/threonine kinase; uPA: urokinase-type plasminogen activator; uPAR, urokinase-type plasminogen activator receptor; CXCR4, C-X-C motif chemokine receptor 4; MT1-MMP, membrane-type 1 matrix metalloproteinase; TNFα, tumour necrosis factor.

The interplay between metastatic markers, MAPKs, and PI3K/AKT pathways reflects the complex nature of cancer progression. The inhibition of metastatic capabilities through downregulation of uPAR, uPA, CXCR4, TNF-α, and MT1-MMP, combined with the altered MAPK and the PI3K/Akt pathway dynamics evidenced by changes in p-IRS, IRS, p-PDK1, p38-MAPK, p-AKT, suggests a comprehensive therapeutic impact. This dual action not only impedes the invasive and migratory potential of cancer cells but also disrupts their metabolic and survival pathways (Figure 5).

For instance, the reduced migratory and invasive capabilities linked to lower uPAR, uPA, and CXCR4 expressions could be further compounded by the disrupted metabolic signalling seen in altered IRS and p-AKT levels [32,33]. This comprehensive impact may lead to reduced proliferation and increased sensitivity to apoptotic triggers, further enhanced by downregulating survival pathways like p-PDK1. The differential responses of MDA-MB-231 and MCF-7 cell lines to PEDF and Dox treatments can be mechanistically understood by considering their distinct biological profiles. MDA-MB-231 cells, being more aggressive and less responsive to hormonal therapies due to their triple-negative status, might rely more heavily on non-hormonal pathways, such as those regulated by CXCR4 and MT1-MMP for metastasis. In contrast, the hormone-sensitive MCF-7 cells might exhibit a more pronounced response to metabolic disruptions, as seen in the alterations in IRS-1 and p-AKT signalling, reflecting their dependence on hormonal pathways for growth and survival.

## 4. Materials and Methods

### 4.1. Reagents

Recombinant PEDF was purchased from MD Bioproducts (Bethesda, MD, USA). The media and supplements were sourced from Sigma-Aldrich and included Dulbecco’s modified Eagle’s medium (DMEM), foetal bovine serum (FBS), antibiotics, and antimycotics. The primary antibodies, including IRS-1, phosphor-IRS1 (tyr 632), Akt (pan) (C67E7), p-Akt (Ser473), p38 MAPK(Thr180/Tyr182), and p-PDK1 (Ser241) inhibitor and secondary anti-rabbit IgG, HRP linked antibody were purchased from Cell Signaling Technology (Beverly, MA, USA). Other antibodies, including CXCR4, MT1-MMP, TNF-α, uPA, and uPAR, were obtained from Santa Cruz Biotechnology (Santa Cruz, CA, USA). Doxorubicin (Dox), trypsin, paraformaldehyde, bovine serum albumin (BSA), saponin, glycerol, Triton X100, dimethyl sulfoxide (DMSO), Tris-ethylenediaminetetraacetic acid (EDTA), eosin, 3,3′-Diaminobenzidine tablets (DAB), and absolute ethanol were from Sigma-Aldrich (St. Louis, MO, USA). Polyclonal goat anti-rabbit biotinylated antibody and normal goat serum (NGS) were purchased from Dako (Mulgrave, VIC, Australia).

### 4.2. Cell Culture

The two human BC cell lines (MCF7; ER/PR-positive and MDA-MB-231; ER, PR, and HER2 negative) were acquired from American Type Culture Collection (ATCC, Manassas, VA, USA) and grown in DMEM containing 10% heat-inactivated foetal bovine serum (FBS) and 1% penicillin/streptomycin. All cultures were maintained at 37 °C in a humidified incubator at 5% CO_2_ and passaged 2 times per week following protocols approved by the ATCC.

### 4.3. Cell Viability

MCF7 and MDA-MB-231 were seeded in 96-well plates at 5 × 10^3^ and 3 × 10^3^ densities, respectively, in four groups: control (without PEDF or Dox); treated with PEDF; treated with Dox; and treated with the combination of PEDF and Dox. The following day, cells were exposed to 100 nM PEDF (physiological concentration of PEDF) [34,35,36] and 0.5 µM Dox [37,38]. Cell viability was assessed via a CellTiter-Blue assay (Promega, Madison, WI, USA). CellTitre-Blue (10 µL) was added to the cells and incubated for 1 h. After that time, the fluorescent signal was assessed at Ex560 nm/Em590 nm with a fluorescence multimode plate reader (Enspire Multimode Plate Reader, PerkinElmer, Waltham, MA, USA). The procedure for each cell line was performed in quadruplicate.

### 4.4. Glucose Uptake

The glucose uptake of MCF7 and MDA-MB-231 was determined using the Amplex^®^ Red reagent (Invitrogen, Waltham, MA, USA) by measuring glucose levels of the corresponding media after 24 h incubation with or without PEDF (100 nM) and Dox (0.5 µM) and the combination of Dox and PEDF. Cells were seeded at the densities of 5 × 10^3^ for MCF-7 and 3 × 10^3^ for MDA-MB-231 in DMEM containing 10% FBS and 1% penicillin/streptomycin. The red fluorescence was detected at Ex571 nm/Em585 nm using the Enspire multimode plate reader (PerkinElmer, Waltham, MA, USA). The glucose consumed over 24 h was calculated by subtracting the glucose concentration at 24 h from that of the complete medium.

### 4.5. Spheroid-Based Migration Assay

To establish tumour spheroid-based migration, MCF7 and MDA-MB-231 cells were dispensed into a ULA (Ultra-Low Attachment) 96-well round-bottomed microplate (Corning) at the densities of 2 × 10^3^ and 1 × 10^3^, respectively. Cells were further cultured for 4 days to allow for the formation of multicellular tumour spheroids possessing a three-dimensional compact structure. Then, tumour spheroids were transferred into a 96-well flat-bottomed plate and were further cultured in the presence or absence of PEDF (100 nM) and Dox (0.5 µM) and the combination of Dox+PEDF. The migration of tumour spheroids was imaged immediately after treatment (0 h) and 24 h using an inverted microscope. Migration inhibition was quantified by measuring the areas of spheroid cells after 24 h covered by migrating cells using ImageJ software (https://imagej.net/ij/).

### 4.6. Immunocytochemistry

ICC experiments and quantitative analysis of staining intensity were conducted as outlined in our previous work [16,39]. Briefly, cells were seeded (MCF-7 at 5 × 10^3^ cells/mL, MDA-MB-231 at 1 × 10^3^ cells/mL) into 96-well plates and incubated overnight. Subsequent treatment involved exposing these cells to PEDF, Dox, Dox+PEDF, and control (no treatment) for 24 h. Post-treatment, cells were fixed using 4% paraformaldehyde and permeabilised with 0.3% saponin. Blocking was then performed using a solution of 2% normal goat or rabbit sera, 0.25% bovine serum albumin (BSA), and 0.1% saponin. Primary antibodies were applied at a 1:500 dilution in phosphate-buffered saline (PBS) overnight. This was followed by a 30-min incubation with secondary antibodies, diluted 1:1000 in PBS. An avidin/biotinylated horseradish peroxidase (HRP) mixture was utilised for signal amplification, following the protocol provided in the ABC kit. Finally, cells were treated with DAB solution, washed, and mounted in 100% glycerol. Imaging was performed using an Olympus inverted IX-51 microscope (Melville, NY, USA) and analysed with CellSens acquisition software v.4.2. Negative control wells were exclusively incubated with PBS to establish a baseline. All observed staining in the experiment was then normalised against these negative controls. This step ensured that the results accurately reflected the specific effects of the treatments by comparing them to a standardised, untreated baseline.

### 4.7. Statistical Analyses

The results of this study are expressed as the mean ± standard deviation. Various statistical methods were employed to determine the significance of the findings, including one-way ANOVA and two-way ANOVA, where applicable. These analyses were conducted using GraphPad Prism version 10 software. A *p* < 0.05 was considered to indicate statistical significance for all assays performed in this study.

## 5. Conclusions

In conclusion, the observations from this study point towards the idea that PEDF may adeptly modulate its activity in response to the Dox-influenced microenvironments, suggesting a dynamic adaptability of PEDF, potentially antagonising or synergising with Dox’s therapeutic effects. Moreover, these results highlight the importance of considering the unique characteristics of different BC subtypes in therapeutic strategies. The differential responses in these two cell lines underlie the potential for personalised and targeted approaches in BC treatment, focusing on specific molecular pathways relevant to each subtype. The combination of PEDF and Dox presents a particularly compelling strategy, effectively targeting both metastatic potential and signalling pathways, which are crucial for cancer cell survival and proliferation. This dual approach may offer a more comprehensive method for tackling the complex nature of BC, potentially improving therapeutic outcomes in these diverse BC subtypes. While we provide evidence of the anticancer effects of PEDF in our cell-based models, future studies in vivo preclinical models are needed as only in such models can the true nature of complex tumours, inclusive of such things as vascularity, immune cells, and progression of a tumour in 3-dimension, be used to truly test the anticancer nature of promising agents such as PEDF.

## Figures and Tables

**Table 1:** Significantly altered metastatic markers among groups in two BC cell lines using a two-way ANOVA and Tukey’s multiple compression tests.

Markers			MDA-MB-231 (^a^ *p*-Values)							MCF-7 (^a^ *p*-Values)		
	PEDF vs. Ctr	Dox vs. Ctr	Dox+PEDF vs. Ctr	PEDF vs. Dox	Dox+PEDF vs. PEDF	Dox+PEDF vs. Dox	PEDF vs. Ctr	Dox vs. Ctr	Dox+PEDF vs. Ctr	PEDF vs. Dox	Dox+PEDF vs. PEDF	Dox+PEDF vs. Dox
**uPAR**	↓ 0.009 **	↓<0.0001 ****	<0.0001	0.0818	↓ 0.007 **	0.7712	0.1236	↓ 0.0151 *	↓ 0.0108 *	0.8155	0.7490	0.9993
**uPA**	↓ 0.0194 *	↓ 0.0046 **	↓ 0.0033 **	0.9538	0.9225	0.9995	↓ <0.0001 ****	↓ <0.0001 ****	↓ <0.0001 ****	0.4310	0.3292	0.9977
**CXCR4**	↓ <0.0001 ****	↓ <0.0001 ****	↓ <0.0001 ****	0.5185	0.2966	0.9783	↓ 0.0002 ***	↓ <0.0001 ****	↓ <0.0001 ****	0.7086	0.2313	0.8282
**MT1-MMP**	↓ <0.0001 ****	↓ <0.0001 ****	↓ <0.0001 ****	↓ <0.0001 ****	↓ <0.0001 ****	0.0528	↓ <0.0001 ****	↓ <0.0001 ****	↓ <0.0001 ****	↓ <0.0001 ****	↓ <0.0001 ****	0.8348
**TNF-α**	↓ 0.0002 ***	↓ 0.0001 ****	↓ <0.0001 ****	0.9983	0.1776	0.2364	↓ <0.0001 ****	↓ <0.0001 ***	↓ <0.0001 ****	↓ 0.0041 **	↓ <0.001 **	0.9438

BC, breast cancer; Ctr, control; PEDF, pigment epithelium-derived factor; Dox, doxorubicin, uPA, urokinase-type plasminogen activator; uPAR, urokinase-type plasminogen activator receptor; CXCR4, C-X-C motif chemokine receptor 4; MT1-MMP, membrane-type 1 matrix metalloproteinase; TNFα, tumour necrosis factor. ^a^ *p*-values are from the two-way ANOVA and the Tukey post hoc test. Arrow (↓) shows down expressions of the markers that changed significantly. (*) *p* ≤ 0.05; (**) *p* ≤ 0.01; (***) *p* ≤ 0.001, and (****) *p* ≤ 0.0001.

**Table 2:** Significantly altered metabolic markers among groups in two BC cell lines using a two-way ANOVA and Tukey’s multiple compression tests.

Markers			MDA-MB-231 (^a^ *p*-Values)							MCF-7 (^a^ *p*-Values)		
	PEDF vs. Ctr	Dox vs. Ctr	Dox+PEDF vs. Ctr	PEDF vs. Dox	Dox+PEDF vs. PEDF	Dox+PEDF vs. Dox	PEDF vs. Ctr	Dox vs. Ctr	Dox+PEDF vs. Ctr	PEDF vs. Dox	Dox+PEDF vs. PEDF	Dox+PEDF vs. Dox
**P-IRS1**	↑ <0.0001 ****	0.8694	0.5480	↑ <0.0001 ****	↓ <0.0001 ****	0.9428	0.1742	↓ 0.0135 *	0.9885	↑ <0.0001 ****	0.0888	↑ 0.0325 *
**IRS-1**	↓ 0.0257 *	↓ 0.0003 ***	↓ <0.0001 ****	0.4634	0.2837	0.9875	↓ <0.0001 ****	↓ <0.0001 ****	↓ <0.0001 ****	↑ 0.0008 ***	↓ <0.0001 ****	0.1379
**p-PDK1**	↓ 0.0003 ***	↓ <0.0001 ****	↓ <0.0001 ****	0.8601	↓ 0.0001 ***	↓ 0.0016 **	↓ <0.0001 ****	↓ <0.0001 ****	↓ <0.0001 ****	↑ <0.0001 ****	↓ <0.0001 ****	0.6118
**P38-MAPK**	↑ <0.0001 ****	0.8148	↑ 0.0217 *	↑ <0.0001 ****	↓ 0.0222 *	0.1727	↑ <0.0001 ****	↑ 0.0269 *	↑ <0.0001 ****	↑ 0.0059 **	↑ <0.0001 ****	↑ <0.0001 ****
**p-AKT**	↓ <0.0001 ****	↓ <0.0001 ****	↓ <0.0001 ****	0.4964	0.2897	0.9823	↓ 0.0045 **	↓ <0.0001 ****	↓ <0.0001 ****	↑ 0.0339 *	↓ <0.0001 ****	↓ 0.0010 ***
**AKT**	0.8869	↓ 0.0286 *	↓ 0.0011 **	0.1554	↓ 0.0103 *	0.6964	0.0603	↑ <0.0001 ****	↑ <0.0001 ****	↓ <0.0001 ****	↑ <0.0001 ****	↓ <0.0001 ****

BC, breast cancer; Ctr, control; PEDF, pigment epithelium-derived factor; Dox, doxorubicin; p-IRS-1*,* IRS-1, insulin receptor substrate 1; PDK1, phosphoinositide-dependent kinase 1; p38-MAPK, p38 mitogen-activated protein kinases; AKT, serine/threonine kinase. ^a^ *p*-values are from the two-way ANOVA and the Tukey post hoc test. Arrows (↑↓) show up/down expression of the markers that changed significantly. (*) *p* ≤ 0.05; (**) *p* ≤ 0.01; (***) *p* ≤ 0.001, and (****) *p* ≤ 0.0001.

## Data Availability

All data generated or analyzed during this study are included in this article.
